# Soluble c-Met Is a Reliable and Sensitive Marker to Detect c-Met Expression Level in Lung Cancer

**DOI:** 10.1155/2015/626578

**Published:** 2015-03-05

**Authors:** Huilai Lv, Baoen Shan, Ziqiang Tian, Yong Li, Yuefeng Zhang, Shiwang Wen

**Affiliations:** ^1^Department of Fifth Thoracic Surgery, Fourth Hospital of Hebei Medical University, Shijiazhuang 050011, China; ^2^Tumor Research Institute, Fourth Hospital of Hebei Medical University, Shijiazhuang 050011, China

## Abstract

c-Met has been demonstrated as an attractive target in lung cancer therapy. Current studies showed that detection of c-Met status in tumor is critical in Met-targeted therapy. However not all patients are suitable for tissue sample collection. It is important to discover novel surrogate markers to detect c-Met status. In the study, soluble c-Met (s-Met) in plasma from 146 Chinese lung cancer patients and 40 disease-free volunteers was measured by enzyme-linked immunosorbent. In parallel, expression of c-Met in those tumors was also assessed by immunohistochemistry. Results showed that, in 146 lung cancer patients, 93 were c-Met expression positive and 74 of 93 were overexpressed. In c-Met-overexpressed patients, plasma s-Met was significantly increased. And further studies showed that plasma s-Met linearly correlated with c-Met expression in tumor. After tumor was removed in Met-overexpressed patients via resection, plasma s-Met significantly decreased to basal level. In addition, plasma s-Met showed to be poorly correlated with tumor size in Met-overexpressed patients. These results demonstrated that plasma s-Met is a sensitive and reliable marker to detect c-Met overexpression in lung cancers, and it is independent of tumor volume.

## 1. Introduction

c-Met is the cell surface receptor for hepatocyte growth factor (HGF) [1]. HGF-induced activation of c-Met results in a complex genetic program referred to as “invasive growth.” It consists of a series of physiological processes including proliferation, invasion, and angiogenesis. It usually occurs in embryonic development, postnatal hepatic, repair of cardiac injury repair and pathologically during oncogenesis [2, 3]. Dysregulation of HGF/c-Met signal axis has been observed in a wide range of human malignancies, including bladder, breast, cervical, colorectal, gastric, head and neck, liver, lung, ovarian, pancreatic, prostate, renal, and thyroid cancer, as well as in various sarcomas, hematopoietic malignancies, and melanoma [4]. In lung cancer, overexpression of c-Met was observed in 40%–60% of patients, and 4% lung cancer patients were found* Met* gene amplification. Furthermore,* Met* gene amplification also was found to be an additional mechanism of acquired EGFR-TKI resistance. Amplified* Met* results in overexpression and overactivation of c-Met and consequently triggers the activation of Her3 which activates downstream signal transducer molecules, such as Akt and Erk, independent of EGFR kinase activity [5]. In clinic, Bean reported that* Met* gene amplification was detected in 22% of acquired EGFR-TKI resistant non-small cell lung cancers, and, compared with patients unexposed to EGFR kinase inhibitor, Gefitinib or Tarceva treatment was more likely to select* Met* gene amplification (21% versus 3%) [1]. The observation provides c-Met as a target in lung cancer therapy. In fact, several Met-targeted molecules are under early clinical evaluation currently. Based on a phase II result of MetMab, a c-Met specific antibody, patients with c-Met overexpression would benefit from Met-targeted therapy, suggesting that detection of c-Met status is critical for Met-targeted therapy. However, not all patients in clinic are suitable for biopsy; thus it is necessary to discover a surrogate marker to detect c-Met status.

Soluble Met (s-Met) is generated via c-Met ectodomain shedding. c-Met is initially synthesized as a single-chain intracellular precursor and subsequently undergoes proteolytic processing at different stages during intracellular trafficking [6], leading to the presentation of an *α*/*β* heterodimer at cell surface. The 140 KD *β* chain of the complex can be proteolytically cleaved by cells constitutively and released to the surrounding environments [7]. s-Met was found to exist in several cancer cells culture supernatants [8], and a significant and direct correlation has been established in preclinical cell line and mouse models between the malignant potential and rate of c-Met ectodomain shedding [9, 10].

Here, we aimed to employ plasma s-Met as a sensitive biomarker to monitor c-Met status in lung cancer tumors and explored the sensitivity and specificity of plasma s-Met in diagnosis.

## 2. Materials and Methods

### 2.1. Patients

Plasma and tumor tissues were obtained from 146 Chinese patients with lung cancer in Fourth Hospital of Hebei Medical University from 2007 to 2012. In parallel, 40 disease-free volunteers were recruited and plasma was collected for reference. In lung cancer patients, 14 were small cell lung cancer (SCLC) and 132 were non-small cell lung cancer (NSCLC). And 47 NSCLC patients received tumor resection; 31 NSCLC patients were diagnosed as EGFR mutation and received EGFR-TKI treatment (erlotinib). The male-to-female ratio was 1 : 1.32, and the median age was 59 years (range from 42 to 83 years). The World Health Organization Classification of Tumor was used to determine histological classification [11]. TNM classification and stage were performed adequately in all patients. All patients provided written informed consent according to the institutional guideline and the study was approved by the institutional review board.

### 2.2. Sample Collection

The tumor tissues were obtained via surgical resection or biopsy. After being fixed in 10% formalin overnight, the tissue fragments were dehydrated by ethanol and embedded in paraffin blocks. The blood was collected before/after treatment, and plasma was separated via centrifugation at 4°C.

### 2.3. Enzyme-Linked Immunosorbent Assay (ELISA)

The concentration of soluble Met in plasma was quantitated using human soluble c-Met quantitative ELISA kit (Invitrogen) which was described in previous study [9]. Briefly, 100 *μ*L of plasma diluent was added into the well precoated with capture antibody and incubated at room temperature for 2 h. Then the medium was removed and the wells were washed with PBST buffer. 100 *μ*L of detection antibody was added and incubated at room temperature for 2 h. The detection antibody was removed and wells were washed with PBST buffer. 100 *μ*L of substrate reagent (R&D, DY999) was added and incubated at room temperature for 20 min. Then 50 *μ*L of 5 M H_2_SO_4_ was added to stop reaction; the density of solution in wells was read at OD_450 nm/570 nm_, respectively. The concentration of s-Met was calculated using standard curve following the suggestion of manufacturer.

### 2.4. Immunohistochemistry (IHC)

Paraffin-embedded tissue blocks were sectioned with 4 *μ*m thickness. Immunohistochemical study was performed using the streptavidin-biotin complex method and TechMate 1000 automated staining system (DakoChemmate, Glostrup, Denmark). Antibody against to c-Met (Roche) was used to detect the expression of c-Met in tumor tissues. The results of staining were evaluated by two independent pathologists who were blind to the clinical data and the difference in interpretation was resolved by consensual agreement. The staining of c-Met in tissues was quantitated using two scoring systems, respectively: (1) the intensity scores: IHC 0 indicated no appreciable staining in the tumor cells; IHC 1+ indicates faint/barely appreciable partial membrane staining in >10% of tumor cells; IHC 2+ indicates weak to moderate staining of entire membrane in >10% of tumor cells; IHC 3+ indicates strong staining of entire membrane in >10% of tumor cells and (2) H scores: H score indicates 0 × (% tumor cells with IHC 0) + 1 × (% tumor cells with IHC 1+) + 2 × (% tumor cells with IHC 2+) + 3 × (% tumor cells with IHC 3+). These scoring systems were also used in previous study [9].

### 2.5. Measurement of Tumor Size

The tumor sizes were assessed using total lengths (TL), which were calculated by summing the lengths of maximum horizontal diameter in major lesions (at most 5 lesions/person). The maximum horizontal diameter of lesion was determined by CT scan.

### 2.6. Statistical Analysis

Chi-square *t*-test and paired *t*-test analysis were used to examine the relationships between groups. Each *P* value was corrected by Bonferroni's method for multiple testing. Prism 5.0 software (GraphPad Software Inc.) was used for statistical analysis. And *P* values less than 0.05 were considered significant.

## 3. Results

### 3.1. s-Met Correlates with Expression Level of c-Met in Lung Cancers

**Figure 1:**
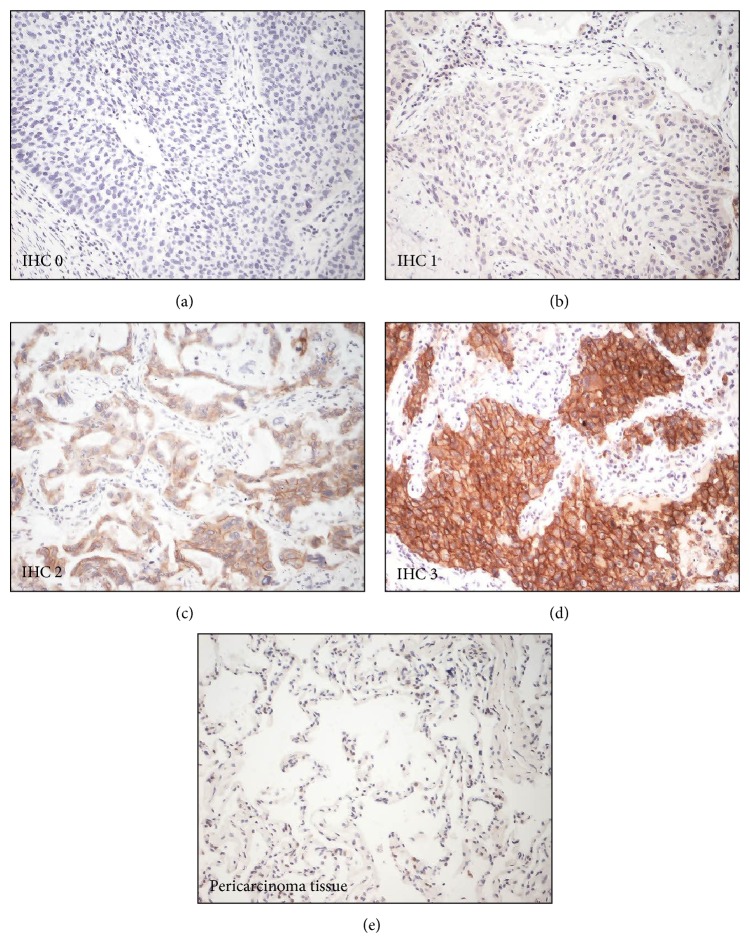
Example cases of c-Met IHC scores. Expression of c-Met in tumors was scored as IHC 0 (a), IHC 1 (b), IHC 2 (c), and IHC 3 (d) based on the staining intensity. And pericarcinoma tissue was used as normal tissue for negative control. No staining of c-Met was detected in pericarcinoma (e).

**Figure 2:**
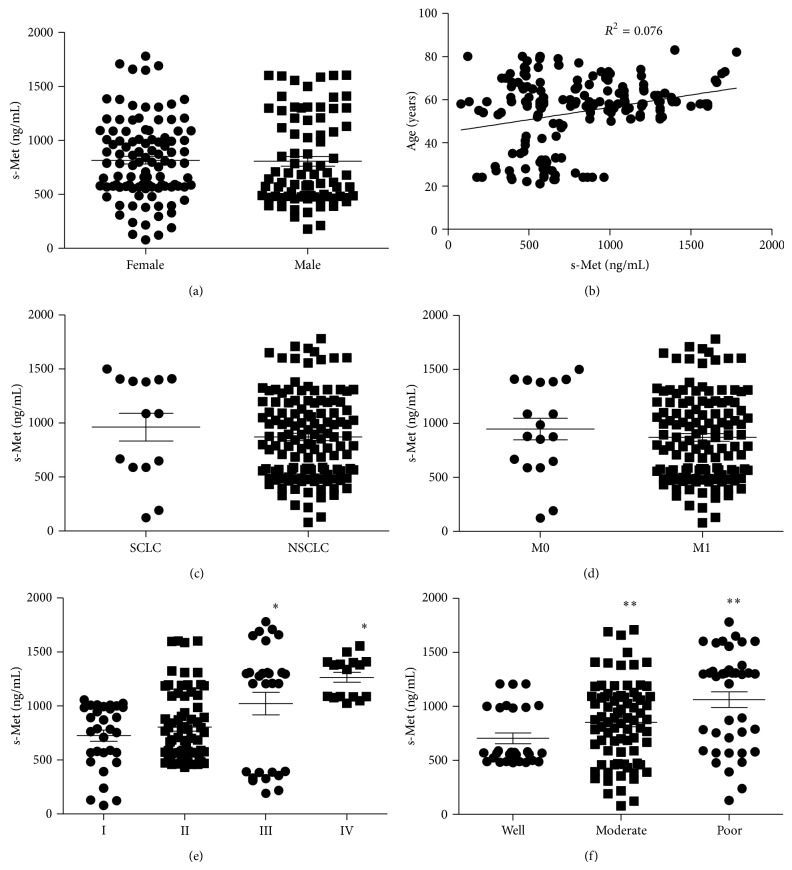
Correlation of plasma s-Met and patients' gender, age, and tumor subtype as well as clinical and histopathological characteristics. s-Met in plasma was measured, respectively, in 146 Chinese lung cancer patients and 40 disease-free volunteers. Individuals were grouped into subgroups according to gender, and s-Met among subgroups was compared (a); correlation of individuals (including patients and disease-free volunteers) age and s-Met (b); s-Met was compared between small cell lung cancer (SCLC) patients and non-small cell lung cancer (NSCLC) patients (c); s-Met was compared between patients with/without metastasis (d); s-Met among patients in various clinical stages was compared; ∗ means significant difference against stage I subgroups, *P* < 0.05 (e); s-Met among tumors with various differentiation was compared; ∗∗ means significant difference against well differentiated subgroups, *P* < 0.05 (f).

**Figure 3:**
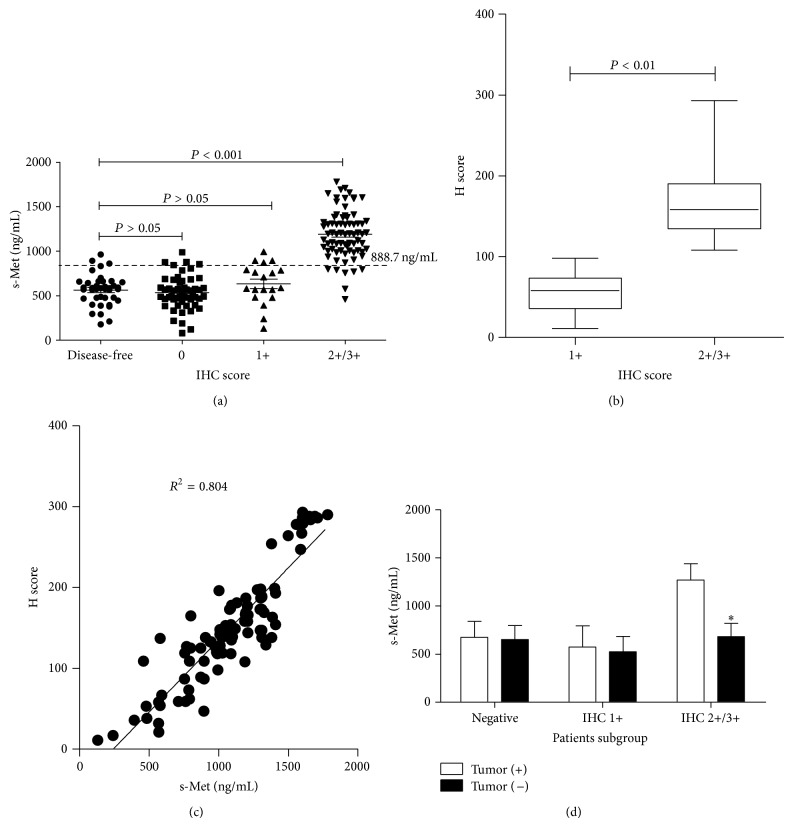
Correlation of plasma s-Met and c-Met expression level in lung cancer patients. s-Met in plasma was measured, respectively, in 146 Chinese lung cancer patients and 40 disease-free volunteers; individuals were grouped into subgroups according to different c-Met expression levels. And s-Met among subgroups was compared (a); correlation of H scores and IHC scores (b); correlation of H scores and s-Met in c-Met expression positive patients (c); changes of s-Met in Met-overexpressed patients after tumors were removed (d). *P* < 0.05 means there is statistical significance.

Expression of c-Met in tumor tissues from 146 lung cancer patients was detected and scored by IHC (Figure 1). Results showed that 93 patients (63.7%) were c-Met expression positive (IHC 1+/2+/3+) and 53 (36.3%) were c-Met expression negative (IHC 0). In Met-positive patients, 74 were overexpressed (IHC 2+/3+). The relationship of c-Met overexpression and histopathological parameters was investigated and results showed that the overexpression of c-Met significantly associated with poorly differentiated, advanced tumor (Table 1). In parallel, plasma s-Met was measured in those lung cancer patients. And 40 disease-free volunteers were recruited and s-Met in their plasma was also measured as reference. Results showed that there was basal level of plasma s-Met in disease-free donors varying among individuals which ranged from 178.5 to 963.0 ng/mL. In lung cancer patients, plasma s-Met showed more variation, ranging from 78.9 to 1781.2 ng/mL. Furthermore, plasma c-Met showed no association with gender (Figure 2(a)), age (Figure 2(b)), lung cancer subtype (Figure 2(c)), and metastasis (Figure 2(d)) but associated with tumor stage (Figure 2(e)) and differentiation (Figure 2(f)), which was consistent with c-Met overexpression. Notably, plasma s-Met correlated well with c-Met expression level. Patients with overexpressed c-Met (IHC 2+/3+) had significantly high plasma s-Met compared with disease-free donors or Met-negative patients (Figure 3(a)) (*P* < 0.05). However, plasma s-Met in patients with weak c-Met expression (IHC 1+) had no significant difference compared with disease-free donors or Met-negative patients (Figure 3(a)) (*P* > 0.05).

In the further studies, c-Met expression level in tissues was evaluated using H scoring system. Results showed that H scores correlated well with the intensity scores. Mean H score in c-Met IHC 2+/3+ patients was significantly higher than c-Met IHC 1+ patients (*P* < 0.01; IHC 2+/3+ versus IHC 1+: 173 ± 54 versus 55 ± 25) (Figure 3(b)). Then H scores were pooled and plotted against plasma s-Met for each Met-positive patient and results were shown in Figure 3(c). As shown in the figure, H scores linearly correlated with plasma s-Met with *R* ^2^ = 0.804, suggesting the good correlation of plasma s-Met and c-Met expression level.

The above results demonstrated that there was basal level of plasma s-Met in disease-free donors, suggesting that, besides tumor, normal tissues also could secret s-Met into blood. In order to confirm whether the increased plasma s-Met in Met-overexpressed patient was from tumor, plasma s-Met in 47 patients who received tumor resection was remeasured 14–20 days after surgery. In those patients, 23 were c-Met-negative, 6 were c-Met IHC 1+, and 18 were c-Met IHC 2+/3+. As shown in Figure 3(d), plasma s-Met significantly decreased in Met-overexpressed patients after tumor burden was removed (*P* < 0.05). In Met-negative patients, removal of tumor did not significantly affect the s-Met level (*P* > 0.05). Furthermore, slight decrease of plasma s-Met was observed in Met-slight patients, but there was no significant difference (*P* = 0.113). The above results demonstrated that overexpression of c-Met in tumor leads to the significant increase of plasma s-Met in blood.

### 3.2. Diagnosis Property of Plasma s-Met

In disease-free subgroup, the upper 95% CI of plasma s-Met was 888.7 ng/mL (Figure 2(a)), which meant that plasma s-Met in 95% of disease-free individuals was lower than 888.7 ng/mL. Setting 888.7 ng/mL as a cut-off to predict the Met-overexpressed positive subjects in population of lung cancer patients and disease-free individuals, results showed that 72 were positive and 114 were negative. In 72 positive objects, 66 were true positive and 6 were false positive. In addition, in 114 negative objects, 106 were true negative and 8 were false negative (Table 2). According to the formulas, (1) sensitivity = [true positive/(true positive + false negative)] and (2) specificity = [true negative/(true negative + false positive)], sensitivity and specificity of plasma s-Met at 888.7 ng/mL in detecting c-Met-overexpressed objects were calculated as 89.2% and 94.6%, respectively (Table 2). These results suggested that plasma s-Met could be a reliable marker to predict c-Met overexpression in tumor tissue.

### 3.3. Plasma s-Met Do Not Correlate with Tumor Size

**Figure 4:**
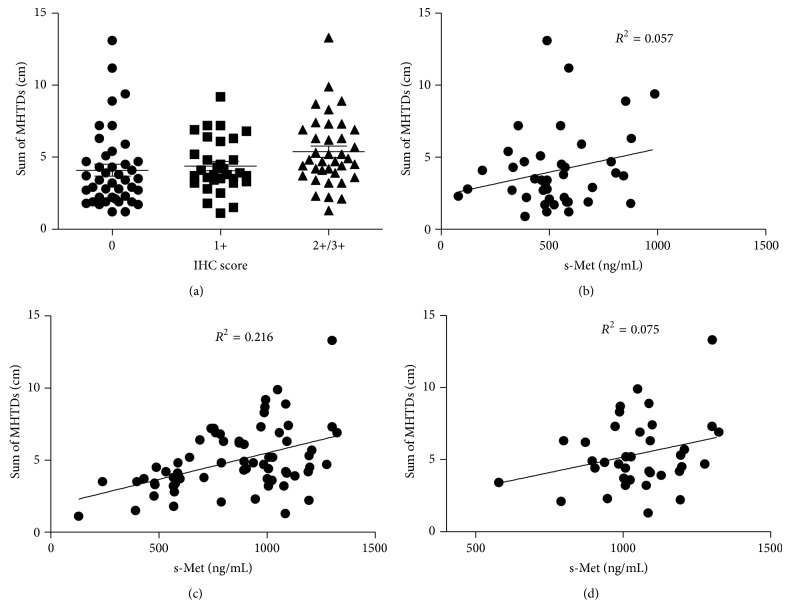
Correlation of s-Met and tumor size. Tumor sizes in patients with different c-Met expression levels were compared (a); s-Met was pooled and plotted against tumor sizes in c-Met expression negative patients (b), c-Met expression positive patients (c), and c-Met-overexpressed patients (d). *P* < 0.05 means there is statistical significance.

Multiple preclinical studies demonstrated that plasma s-Met directly correlated with tumor size in xenograft mice [9]. In order to explore the relationship of plasma s-Met and tumor size in human, we measured tumors' size in 97 patients with local primary tumor either alone or in conjunction with metastases lesions. The size of each lesion (including primary and metastatic lesion) was determined using the maximum horizontal tumor diameter (MHTD) obtained from CT scan, which is described in the previous study [12]. And lesions with MHTD smaller than 10 mm would be excluded from evaluation. The tumor size was calculated by summing total MHTDs of lesions. In evaluable 97 patients, 45 were c-Met-negative, 12 were c-Met IHC 1+, and 40 were Met-overexpressed. The mean tumor size is 4.6 ± 2.4 (cm), ranging within 1.1~13.3 cm. As shown in Figure 4(a), there was no significant difference of tumor size among patients with various c-Met expression levels. Although tumors size in c-Met-overexpressed patients slightly increased, there was no statistical significance (*P* > 0.05) compared with c-Met-negative or weakly expressed patients. In order to further evaluate the correlation of plasma s-Met and tumor size, plasma s-Met concentrations were pooled and plotted against tumor sizes, and calculated results are presented in Figures 4(b), 4(c), and 4(d). Results showed that plasma s-Met correlated with tumor size neither in Met-negative patients (Figure 3(b)) nor in c-Met-positive patients (IHC 1+/2+/3+) (Figure 3(c)). Even in c-Met-overexpressed patients (IHC 2+/3+), plasma s-Met poorly correlated with tumor size (Figure 3(d)).

## 4. Discussion

Plasma s-Met was generated by shedding of c-Met ectodomain, which was regulated through broadly distributed signaling pathways including those of EGF, G-protein coupled receptors, and integrins as well as intracellular pathways activated by phorbol myristate acetate [7, 13]. Preclinical research revealed that shedding rate of c-Met correlated with malignant potential of xenograft tumors with c-Met overexpression [14], which brought plasma s-Met as a potential surrogate marker in human. Relationship between plasma s-Met and malignances has been studied in clinic, but data were limited and contradictory. Result from a phase 2 study of AMG102, an anti-HGF antibody, demonstrated that plasma s-Met in serum decreased after drug treatment, but it did not correlate with outcomes [15]. Sorbellini reported that plasma s-Met level in urinary correlated with bladder cancer stages [16]. However, Wader reported that there was no difference in plasma s-Met between healthy volunteers and myeloma patients, but c-Met status in these myeloma patients was not examined [17]. In this study, we measured plasma s-Met in 40 disease-free volunteers. Although c-Met is not expressed in most of the organs in adult, it is still considerately expressed in endothelial cells, neurons, hepatocytes, hematopoietic cells, and melanocytes [18]. And, theoretically, plasma s-Met would be released from these cells and would be secreted into blood, which made it maintain at in a basal level. As our prediction, plasma s-Met was detected in disease-free volunteers at various ranges of concentrations among different individuals. In lung cancer patients, plasma s-Met was detected at wider concentrations span. And plasma s-Met levels correlated with expression level of c-Met; Met-overexpressed patients harbored significantly higher plasma s-Met. Furthermore, when tumor burden was removed in these patients, plasma s-Met significantly decreased to low level which was close to that in disease-free volunteers, suggesting that overexpression of c-Met in tumor tissue leads to the significant increase of plasma s-Met. And our results showed that plasma s-Met in 95% of disease-free volunteers was lower than 888.7 ng/mL. Using the concentration as a criterion to predict c-Met overexpression in patients, the sensitivity and specificity were 89.2% and 94.6%, respectively. These results showed that plasma s-Met could work as a sensitive surrogate marker to detect c-Met overexpression in lung cancers. Although our results provided 888.7 ng/mL as a criterion to diagnose c-Met overexpression, the data were from limited sample size and need to be further explored in larger population. In addition, our results showed that increased plasma s-Met was independent of tumor volume, suggesting it would not likely work as a pharmacodynamic marker in further Met-targeted therapy. Taken together, plasma s-Met is a reliable and sensitive enough marker to detect c-Met overexpression in lung cancers.

Although multiple preclinical studies demonstrated that plasma s-Met correlated well with tumor volumes in mice bearing Met-positive xenograft tumor and Fu et al. also reported that plasma s-Met associated with tumor size in NSCLC [9, 10], in the present study, we observed that plasma s-Met poorly correlated with tumor size. We think the reasons which made the observations different were due to the following. (1) In xenograft tumor, expression of c-Met was homogeneous. Although correlation of s-Met and tumor volume was observed, in fact it reflected the correlation of s-Met and total c-Met amount in tumor which was confirmed by Fu's investigation [9]. However, in human tumor, expression of c-Met was heterogeneous and there was no correlation of c-Met expression and tumor volume. (2) In Fu's results, author observed that plasma s-Met among patients with various tumor sizes was different, but it was a rough comparison and did not exclude the impact of various c-Met expression levels among those subgroups on plasma s-Met, which may lead to false positive conclusion. Our observation revealed that plasma s-Met seemingly correlated well with later staged and poorly differentiated tumors, but it actually reflected the correlation of c-Met expression and tumor status, which was due to the good correlation of plasma s-Met and c-Met expression level.

Overexpression of c-Met has been documented as a prognostic marker in non-small cell lung cancers, which associated with poorer outcome of NSCLC [19]. Although preclinical data suggested that* Met* gene amplification may be considered as a prognostic marker [20], however, results from a phase 2 study of an anti-c-Met antibody, MetMab, demonstrated that only expression of c-Met rather than* Met* gene amplification was a sensitive and independent predictor [21]. Thus determination of c-Met expression level was critical in Met-targeted therapy. However, currently, detection of c-Met depended on the obtainment of tissue sample via biopsy or surgery, which was different in clinical practices and not all patients were suitable for tissue sampling. Furthermore, heterogeneous expression of c-Met in tumor would affect overall evaluation of c-Met status in whole tumor based on limited tissue blocks from focal areas. Thus, discovery of surrogate markers which could sensitively reflect the c-Met expression level in tumor was important and it would increase the efficiency of patient selection. In addition, dynamic detection of c-Met was also important in treatment. Our result showed that plasma s-Met was a sensitive and specific enough surrogate marker to detect c-Met status in tumor. For instance, it was well known that* Met* gene amplification was an important mechanism which acquired resistance in EGFR-targeted therapy. Because plasma s-Met was easily measured, it could be used to detect dynamic changes of c-Met in tumor.

## 5. Conclusion

In conclusion, plasma s-Met would function as a prognostic marker in lung cancers; it correlated well with c-Met expression in tumor but did not associate with tumor size. Plasma s-Met over than 888.7 ng/mL could specifically predict the c-Met overexpression in lung cancer.

## Figures and Tables

**Table 1:** Clinical and pathological characteristics of lung cancer patients with c-Met overexpression.

Feature	Patients (*n*)	(%)	c-Met OE (*n*)	%	*P* value
Gender					
Male	63	43.2	30	47.6	>0.05
Female	83	56.8	44	53.0	
Histopathological subtypes					
SCLC	14	9.6	7	50.0	>0.05
NSCLC	132	90.4	67	50.8	
Clinical stage					
I	32	21.9	11	34.4	<0.05
II	70	47.9	29	41.4	
III	28	19.2	20	71.4	
IV	16	11.0	14	87.5	
Lymph node					
Negative	102	69.9	50	49.0	>0.05
Positive	44	30.1	24	54.5	
Metastasis					
M0	128	87.7	65	50.8	>0.05
M1	18	12.3	9	50.0	
Differentiation					
Well	28	19.2	9	32.1	<0.01
Moderate	79	54.1	33	41.8	
Poor	39	26.7	32	82.1	

SCLC: small cell lung cancer; NSCLC: non-small cell lung cancer; OE: overexpression.

**Table 2:** Sensitivity and specificity of s-Met at 888.7 ng/mL in predicting Met-overexpressed patients.

Total	Positive (*n*)			Negative (*n*)			Sensitivity (%)	Specificity (%)
	Predicted	True	False	Predicted	True	False		
186	72	66	6	114	106	8	89.2	94.6
